# A novel c.-22T>C mutation in *GALK1 *promoter is associated with elevated galactokinase phenotype

**DOI:** 10.1186/1471-2350-10-29

**Published:** 2009-03-24

**Authors:** Hyung-Doo Park, Yoon-Kyoung Kim, Kyoung Un Park, Jin Q Kim, Young-Han Song, Junghan Song

**Affiliations:** 1 Department of Laboratory Medicine, Samsung Medical Center, Sungkyunkwan University School of Medicine, Korea; 2 Ilsong Institute of Life Science, Hallym University, Anyang 431-060, Korea; 3 Department of Laboratory Medicine, Seoul National University Bundang Hospital, 300 Gumi-dong, Bundang-gu, Gyeonggi-do 463-707, Korea; 4 Department of Laboratory Medicine, Seoul National University College of Medicine, Korea

## Abstract

**Background:**

Many genetic variations of *GALK1 *have been identified in the patients with galactokinase (GALK1) deficiency. However, the molecular characteristics of *GALK1 *in individuals with elevated GALK1 activity are relatively unknown.

**Methods:**

We investigated the relationship between elevated GALK1 activity and the molecular *GALK1 *gene variations, and the molecular mechanism underlying elevated GALK1 activity. PCR products from 63 subjects, without any attenuation of galactose degradation enzymes, were sequenced to screen for nucleotide alterations in the *GALK1 *promoter.

**Results:**

Three nucleotide substitutions were identified: c.-179A>G, c.-27A>C, and c.-22T>C. With respect to the c.-22T>C mutation, GALK1 activity in 13 subjects with the T/C or C/C genotype was significantly higher than those in 50 subjects with the T/T genotype (p < 0.001). The dual luciferase reporter assay in Hep3B cells showed that the luciferase activity with the *GALK1 *promoter with the c.-22C mutant allele increased approximately 2.5-fold, compared to that with the c.-22T. A specific DNA-protein complex was observed in an electrophoretic mobility shift assay, with slightly higher affinity to c.-22C than to c.-22T.

**Conclusion:**

The c.-22T>C mutation, which was observed frequently in individuals with elevated GALK1 activity, increased the expression of a reporter gene through enhanced binding of a currently unidentified nuclear protein. These results suggest that the elevated GALK1 activity resulted from enhanced gene expression, due to nucleotide variation within *GALK1 *promoter.

## Background

Galactose is converted into glucose-1-phosphate by the action of three enzymes: galactokinase (GALK1: EC 2.7.1.6), galactose-1-phosphate uridyltransferase (GALT: EC 2.7.7.12), and UDP-galactose-4'-epimerase (GALE: EC 5.1.3.2). Galactosemia (MIM 230400, 230350 and 230200) is an autosomal recessive disorder that results from defects in one of the above enzymes and that affects a patient's ability to appropriately metabolize galactose [1]. The symptoms and severity of the disease vary, depending on the affected enzyme and the degree of functional defects.

Newborn screening tests for galactosemia have been performed in Korea as well as in many developed countries. Abnormally low GALT, GALE, or GALK1 activities result in a positive newborn screen; however, a positive galactosemia screening does not indicate only the possibility of enzyme deficiency, but is perhaps as a reflection of portosystemic shunt or liver dysfunction, e.g. citrin deficiency and Fanconi-Bickel syndrome [2].

Previously, we investigated the phenotypic distribution of 249 Korean patients with a positive newborn screen for galactosemia and found that elevated GALK1 activity was observed frequently in these newborns [authors' unpublished data]. In patients with the GALK1 deficiency, various nucleotide changes in the coding region of the *GALK1 *gene have been identified [3-9]. However, no study has been performed with regard to the molecular characteristics of *GALK1 *in individuals with increased GALK1 activity. The exact incidence of GALK1 deficiency is unknown but is probably 1:1,000,000 [1,4], and the incidence with elevated GALK1 activity has not been reported.

Interestingly, the enzyme activity of GALK1 changes during development: GALK1 activity in newborns is distinctly high (80–120 nmol/min/g Hb) and decreases sharply with age until they are one year old (20–30 nmol/min/g Hb)[10,11] These low values are maintained for the remainder of life. The *GALK1 *promoter region is shown to have high GC content, with several binding sites for the Sp1 transcription factor, and the absence of TATA-box and CCAAT-box [12]. During the development of suckling mouse liver, Egr-1, a transcription factor, was reported to regulate GALK1 transcription [13]. The presence of putative binding sites for Egr-1 and Sp1, in the promoter region of human *GALK1 *gene, prompted us to speculate that an increased GALK enzyme activity might be caused by the transcriptional activation of the *GALK1 *promoter.

To investigate the relationship between elevated GALK1 activity and transcriptional regulation of *GALK1 *gene, we analyzed sequence variations in the promoter region of the *GALK1 *gene, in individuals with elevated GALK1 activity. In addition, we speculate that elevated GALK1 activity could be one of the possible causes for a positive result for galactosemia in newborn screening.

## Methods

### Subjects

In Korea, individuals with a positive result in the neonatal total galactose screening test performed by colorimetric enzyme assay on dried blood spots, were tested for GALK1, GALT, and GALE enzyme activities, as well as for galactose-1-phosphate levels in erythrocytes. Some individuals showed decreased enzyme activities of GALT, GALE, and/or GALK1, and these were investigated for the molecular characteristics of their coding region [9,14]. Sixty-three subjects without any attenuation in galactose degradation enzymes and with a median age of 29 days were selected for the present study. Total galactose level by newborn screening assay on dried blood spots, for all subjects, was 15.3 ± 8.0 mg/dL (mean ± SD, 8.1 – 40.0). GALK1, GALT, and GALE activities in red blood cells derived from heparinized whole bloods, were measured by radiometric assays, which were based on the formation of radio-labeled galactose-1-phosphate from [^14^C]galactose and ATP, radio-labeled UDP-galactose from [^14^C]galactose-1-phosphate and UDP-glucose, and radio-labeled UDP-glucose from [^14^C]UDP-galactose (described elsewhere), respectively [11]. Galactose-1-phosphate levels in red blood cells were also measured by radiometric assay [11]. This research was approved by the Institutional Review Board of the Seoul National University Bundang Hospital.

### PCR and direct sequencing

Human genomic DNA was isolated from frozen white blood cells, using a HighPure Viral Nucleic Acid Isolation Kit (Roche Diagnostics, USA). PCR was performed on genomic DNA to amplify the promoter region (nucleotide -558 to +71, the translation start site is designated as +1.) of the *GALK1 *gene. To cover the promoter region of the *GALK1 *gene, two overlapping regions, GKP1 and GKP2, were amplified using two sets of primers: GKP1-F (5'-CCG GCC CAA ACT TGT CTC TG-3') and GKP1-R (5'-GTG GCA GGG GCT AAT GGT G-3'), GKP2-F (5'-TGG TTC TTC CCG AAG TCC AG-3') and GKP2-R (5'-AAC TCC TCC CGG AAG GCT C-3'). Up to 50 μL of master mixture was made, containing 300 ng of genomic DNA, 10 pM of each primer, 1× PCR buffer, 0.2 mM dNTPs, and 1.25 U Taq polymerase, for PCR of the first promotor region. For the second promoter region, 25 mM MgCl_2 _was added to the same PCR condition of the first promoter region of *GALK1*, with 0.625 U Taq polymerase included. After an initial denaturation step, at 94°C for 5 min, 30 cycles of amplification were carried out using the following procedures: denaturation at 94°C for 30 sec, annealing at 61°C for 30 sec, extension at 72°C for 30 sec (for the amplification of GKP1 promoter region or denaturation at 94°C for 1 min), annealing at 56°C for 1 min, and extension at 72°C for 1 min for GKP2, respectively. A final extension step at 72°C for 5 min also was added. The PCR products were purified using an ExoSAP-IT (USB corp., OH, USA) and sequenced using an ABI Prism^® ^BigDye™ Terminator Cycle Sequencing Ready Reaction Kit v3.1 protocol. Data were analyzed using an ABI3730XL DNA sequencer (Applied Biosystems, USA).

### PCR and RFLP (restriction fragment length polymorphism)

To confirm the presence of the polymorphisms identified by sequencing, RFLP was performed on the PCR products. *Bsg*I (New England Biolabs, MA, USA) restriction enzyme was used to confirm the c-22T>C mutation on the PCR products, using the primer pair, GKP2-F and GKP2-R, which was the same as used for sequencing. However, *Pvu*II (New England Biolabs, MA, USA) GKP2-R' (5'-CGT GCA GCC CCT CAC CAT AG-3') primer was used to confirm the c.-179A>G and c.-27A>C mutations. We screened 101 healthy adults without any clinical symptoms of galactosemia to exclude common nucleotide polymorphism.

### Dual luciferase reporter assay

For this study, the promoter region of the human *GALK1 *gene (-441 to -1) was cloned into the pGL3-basic firefly luciferase vector (Promega Corp., WI, USA). Nucleotides c.-179A, c.-27A, and c.-22T were mutagenized to G, C, and C, respectively, using a QuikChange Site-directed Mutagenesis kit (Stratagene, CA, USA). All constructs were confirmed by nucleotide sequencing of the entire promoter region, and all components for cell culture were purchased from JBI (Wel Gene Inc., Korea). Hep3B and HepG2 cells were grown in Dulbecco's modified Eagle medium (DMEM), supplemented with 10% fetal calf serum, penicillin G (100 units/mL), and streptomycin sulfate (100 mg/mL). Cells grown in a 60-mm dish were transfected with plasmids (5 μg of GALK1 promoter in pGL3-basic and 0.5 μg of pRL-TK vector) using calcium-phosphate. Cell lysates were prepared and assayed for luciferase activity, according to the manufacturer's instruction, using the dual luciferase reporter assay system (Promega Corp., WI, USA). The assays were performed five times in duplicate, and the data were normalized by *Renilla *luciferase luminescence intensity.

### EMSA (electrophoretic mobility shift assay)

Nuclear extracts were prepared as previously described [15,16], except that all solutions contained 1 mM sodium vanadate, 1 mM sodium fluoride, and 1× commercial protease inhibitor cocktail (Boehringer Mannheim Corp., Indianopolis, IN). Radio-labeled oligonucleotide probes [*GALK1*22T (5'-CTG TGC CGG AGC AGC TGT GCA GAG CTG CAG GCG-3') and *GALK1*22C (5'-CTG TGC CGG AGC AGC TGC GCA GAG CTG CAG GCG-3')] were prepared as follows: the sense and complementary antisense oligonucleotides used as probes were synthesized, HPLC purified, and then annealed in 100 mM NaCl and 50 mM Tris-HCl pH 7.5, by heating to 90°C for 3 min, and then cooling to 25°C at 1°C, per 3 min, in the thermal cycler. Annealed oligonucleotides were end-labeled using T4 polynucleotide kinase and [γ-^32^P]ATP, by incubation at 37°C for 30 min. Unincorporated radioactive nucleotides were removed by the QIAquick Nucleotides Removal Kit (QIAGEN). Four μg of Hep3B nuclear extract was incubated with 20,000 cpm of the labeled probe in a reaction mixture containing 10 mM HEPES pH 7.9, 100 mM KCl, 0.5 mM EDTA, 100 mM KCl, 6 mM DTT, 5% glycerol, and 2 μg of poly(dI-dC):poly(dI-dC) (Pharmacia Biotech Inc., Piscataway, NJ). The binding mixture was incubated at room temperature for 20 min and run on a 6% nondenaturing polyacrylamide gel in 1× TBE buffer at 10 V/cm. The gel was dried at 80°C, under vacuum, and exposed to an X-ray film. Competition assay was performed with unlabeled competitor DNA with c.-22C or c.-22T. Supershift analysis was performed using anti-HEN1 antibody (Chemicon International). MatInspector [17] was used to identify the transcription factors that might bind to the GALK1 promoter.

### Statistical analysis

To standardize GALK1 activity according to patient age, we obtained the normalized GALK1 value, by dividing measured GALK1 activity by calculated GALK1 normal value. Calculated GALK1 normal activity was obtained according to patient age (less than 6 months) using the following equation; y = 0.0024x^2 ^- 0.7868x + 105.72 (where x is age in days and y is GALK1 normal activity). The above quadratic equation was derived from 146 subjects with normal GALK1 activity, and the data are reported as the mean ± SD (standard deviation). In individuals with different genotypes, GALK1 activities, their normalized values, and galactose-1-phosphate levels were compared using the Mann-Whitney U test. The Spearman correlation analysis was used to test for unadjusted association between GALK1 activities and galactose-1-phosphate levels, with statistical significance set at a p value of 0.05. All analyses were performed using the SPSS 12.0 K for Windows (SPSS Inc., Chicago, IL, USA).

## Results

### Mutations in the GALK1 promoter region

**Figure 1:**
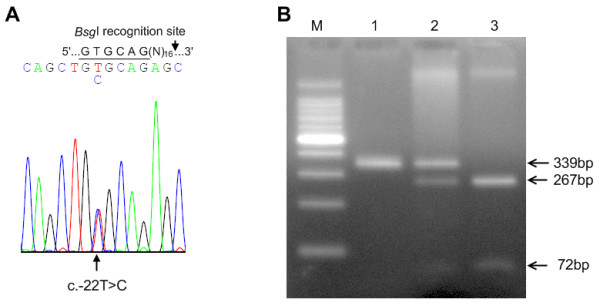
**Identification of sequence variations in the *GALK1 *promoter region**. Sequencing revealed the presence of a mutation of c.-22T>C (A). Gel electrophoresis patterns of PCR amplified DNA fragments digested with *Bsg*I for the confirmation of c.-22T>C (B) are shown (Lane M, DNA size marker; lane 1, C/C; lane2, T/C; lane 3, T/T). *Bsg*I recognizes the sequence GTGCAG (underlined in A electrophoretogram) present in wild type c.-22T, but not in c.-22C. The PCR fragment from genotype c.-22C/C, c.-22T/C and c.-22T/T will produce (à produce) one (339 bp), three (339, 267 and 72 bp) and two (267 and 72 bp) bands.

Sixty-three Koreans with positive newborn screening results, who did not have any attenuation in galactose degradation enzymes, were studied. GALK1 activity, for all subjects, was 113.1 ± 41.5 nmol/min/gHb (mean ± SD, 50.2 – 243.3). We analyzed the underlying molecular defects of the *GALK1 *gene using PCR and direct sequencing. Three nucleotide substitutions were identified in the promoter region of the *GALK1 *gene: c.-179A>G, c.-27A>C, and c.-22T>C (Fig. 1), and the presence of these polymorphisms was confirmed by performing PCR-RFLP (Fig. 1). For c.-179A>G, 56 subjects with A/A genotype, 5 with A/G genotype and 2 with G/G genotype were identified. For c.-27A>C, 61 individuals had A/A genotype and 2 showed A/C genotype. Various genotypes at nucleotide position c.-179 (A/A, A/G, and G/G) and at c.-27 (A/C and A/A) were also present in 101 normal individuals, suggesting that two SNPs, c.-179A>G and c.-27A>C, are benign sequence variations. In terms of c.-22T>C, 50 individuals with T/T, 12 with T/C, and 1 with C/C genotype were identified (Table 1). Every individual in the control group showed the T/T genotype at position c.-22, which suggests that T to C substitution may be a mutation, rather than polymorphism. Genotype distributions of the c.-179A>G, c.-27A>C and c.-22T>C variations did not deviate from those expected for Hardy-Weinberg proportion.

**Table 1:** Genotype distribution of three nucleotide variations in the promoter region of *GALK1 *gene and their galactokinase values in 63 subjects

Nucleotide substitution	Genotype	No.	Galactokinase value (nmol/min/gHb)		Normalized GALK1 value	
					
			mean ± SD	*p *value*	mean ± SD	*p *value*
c.-179A>G	A/A	56	117.2 ± 41.7	0.012	1.39 ± 2.06	0.001
	A/G	5	82.6 ± 23.2		0.99 ± 0.13	
	G/G	2	73.8 ± 11.8		0.78 ± 0.10	
						
c.-27A>C	A/A	61	112.1 ± 41.7	0.173	1.34 ± 0.46	0.473
	A/C	2	141.4 ± 27.4		1.47 ± 0.27	
	C/C	0	-		-	
						
c.-22T>C	T/T	50	96.1 ± 24.3	<0.001	1.16 ± 0.25	<0.001
	T/C	12	176.9 ± 26.9		2.06 ± 0.30	
	C/C	1	193.6		2.30	

*p *value was determined by SPSS 12.0 by Mann-Whitney test

**Figure 2:**
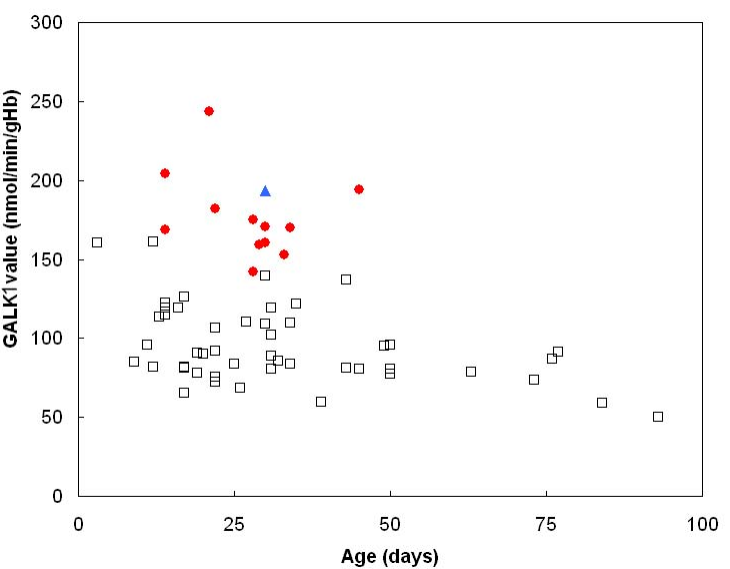
**Distributions of GALK1 activities according to the genotypes at c.-22 of the *GALK1 *gene are plotted according to patient age**. The GALK1 values for the genotype C/C, T/C, and T/T at nucleotide -22 are indicated as ▲, ●, and □, respectively. The GALK1 activities in individuals having at least one C allele (T/C or C/C genotype) were significantly higher than those in individuals with the T/T genotype (*p *< 0.001 by Mann-Whitney U test).

In terms of c.-22 of *GALK1*, GALK1 activities, and their normalized values in individuals having at least one C allele (T/C or C/C genotype), were significantly higher than those in individuals with T/T genotype (*p *< 0.001 by Mann-Whitney U test, respectively) (Table 1 and Fig. 2). There were statistical differences in GALK1 activities between subjects with A/A, and those with A/G or G/G, at c.-179 of the *GALK1*; however, it disappeared after excluding 13 individuals with the C allele at c.-22 from total subjects. GALK1 activities in 43 individuals with A/A, versus in 7 individuals with A/G or GG genotype at position c.-179 of *GALK1*, were 98.7 ± 24.1 versus 80.1 ± 20.0 nmol/min/gHb (*p *= 0.307). This might be because the C allele at c.-22 is linked closely with the A allele at c.179. On the contrary, there was no statistical difference in GALK1 activities between the subjects with A/A and A/C genotypes at position c.-27 of *GALK1 *(Table 1), which further suggests that c.-27A>C is a nonfunctional polymorphism.

Elevated GALK1 activity could result from increased protein level or from the hyperactivity of the enzyme, without changes in protein abundance due to amino acid alteration. To test this alternative, we sequenced the PCR product of all coding exons of the *GALK1 *genes for 13 individuals having the C allele at nucleotide -22, according to the method described elsewhere [9]. There was no evidence of nucleotide changes in the coding regions of *GALK1*, in the individuals with T/C or C/C genotypes, suggesting that the elevated GALK1 activity is due to increased GALK1 expression.

To investigate whether elevated GALK1 activity resulting from -22T>C mutation is associated with an increase of galactose-1-phosphate in newborns, we measured galactose-1-phosphate levels. Individuals having at least one C allele (n = 13, 1.59 ± 1.81 mg/dL) showed significantly higher galactose-1-phosphate levels than did the individuals with c.-22T/T (n = 50, 0.94 ± 1.50 mg/dL) (*p *= 0.019 by Mann-Whitney U test).

### Promoter Analysis of GALK1 Gene

**Figure 3:**
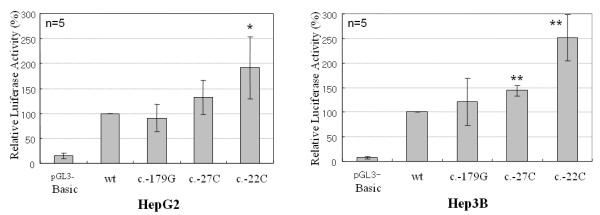
**c.-22T>C mutation resulted in increased reporter activity in hepatoma cell lines**. The promoter region of the *GALK1 *gene was cloned into pGL3-Basic, and the nucleotides at positions c.-179, c.-27, and c.-22 are indicated. Each construct was transfected into HepG2 and Hep3B cells five times in duplicate, and dual luciferase assays were performed. The mean luciferase activities, normalized for cell transfection efficiencies, were calculated relative to the activity of the wild type construct (wt, set as 100). Standard deviations are indicated. *p < 0.05 and **p < 0.01, significantly different between transfected cell lines and wild type by paired t-tests.

To test if c.-22T>C affected transcription, reporter analysis was performed using the promoter region of *GALK1 *gene (-441 to -1), with nucleotide substitutions at position -179, -27, and -22. The HepG2 cells transfected with the c.-22C mutant allele showed an elevated luciferase activity approximately two-fold over those with the wild type sequence. This phenomenon appeared more clearly in the Hep3B cells showing a two and a half fold increase (Fig. 3). In terms of c.-179A>G and c.-27A>C, the relative luciferase activity was not significantly different from that of the wild type.

**Figure 4:**
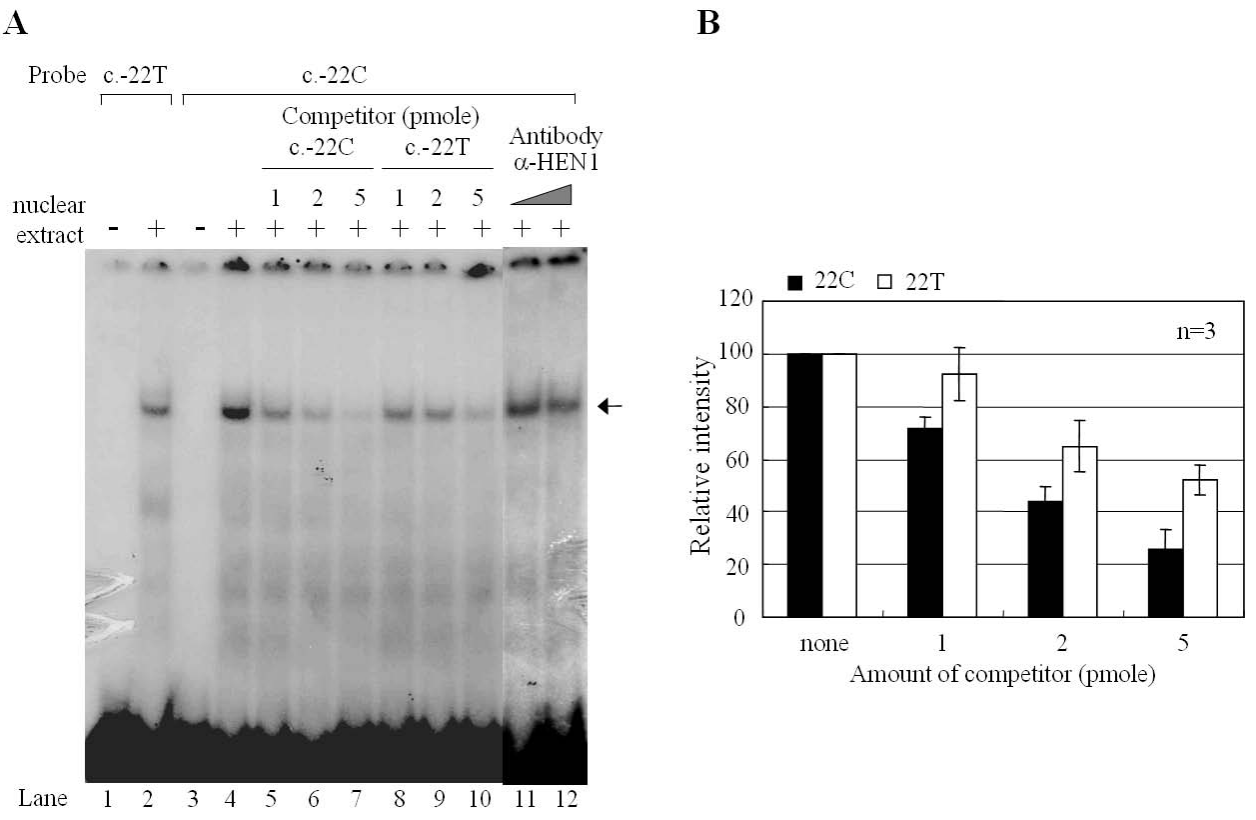
**Oligonucleotide encompassing c.-22 formed DNA-protein complex detected by electrophoretic mobility shift assay (EMSA)**. **A) **The radiolabeled oligonucleotide probes containing c.-22T or c.-22C of the *GALK1 *gene were incubated with Hep3B nuclear extract. When c.-22C was used as a probe, increasing amounts of cold competitors (1, 2, and 5 pmol of c.-22C and c.-22T) or anti-HEN1 antibody were included as indicated. The DNA-protein complex is indicated with an arrow. **B) **The intensity of the DNA-protein complex in the absence and presence of cold probes, c-22T (□) and c.-22C (■), was measured using ImageJ. The graph represents means and standard deviations of three independent experiments taking the intensity of DNA-protein complex without competitor as 100%.

EMSA was performed to determine if the elevated luciferase activity of c.-22T>C was due to alterations in the binding of a nuclear protein. Thirty-three-bp oligonucleotides with c.-22T and c.-22C were used as a probe, and incubation of these radio-labeled probes with Hep3B nuclear extracts produced a DNA-protein complex (Fig. 4A lanes 2 and 4). When the c-22C DNA-protein complex was challenged with increasing amounts of cold probes, the competition was slightly more efficient with c-22C, than with c-22T (Fig. 4A). The competition analysis was performed three times and the relative band intensity of the DNA-protein complex was consistently higher when competed with c-22T than with c-22C (Fig 4B). This result suggests that the Hep3B nuclear proteins interacted with the c-22C oligonucleotides with slightly higher affinity than with the c-22T. MatInspector was utilized to identify a transcription factor that might differentially interact with c-22C and c-22T, and HEN1 was identified as a potential candidate. However, addition of anti-HEN1 antibody did not affect the mobility of the DNA-protein complex (Fig 1A, lanes 11 and 12). In addition, no supershift was observed with anti-Egr1 and anti-Sp1 antibodies (data not shown).

## Discussion

The actions of GALK1 and GALT maintain the level of galactose-1-phosphate. The increase of galactose-1-phosphate, by an abnormal metabolic pathway, may cause various problems, as galactose-1-phosphate is thought to be a toxic metabolite [18]. Classic galactosemia is a representative disease of an accumulation of galactose-1-phosphate, by GALT deficiency, which is known to be the most common cause of galactosemia [19]. Previously, we observed elevated GALK1 activity was common in Korean newborns with a positive neonatal screen result for galactosemia. Unlike GALK1 deficiency, little is known about the molecular mechanisms that lead to an increase in GALK1 enzyme activity. In this study, we analyzed 63 newborns with elevated GALK1 activity but without attenuated GALT and GALE activities. We found that the nucleotide change c.-22T>C in the promoter region of GALK1 gene was highly correlated with elevated GALK1 activity and galactose-1-phosphate levels possibly by increasing gene expression.

The luciferase reporter construct containing -441 to -1 of the *GALK1 *promoter was able to support the expression of the reporter gene in hepatoma cell lines, HepG2 and Hep3B, suggesting that this region has promoter activity. The reporter construct with c.-22T>C enhanced luciferase activity, both in the HepG2 and Hep3B cells, where the fold increase was similar to GALK1 activity seen in the subjects with the C allele (Table 1). In conjunction with the EMSA result, this data suggests that the c.-22T>C resulted in an increased abundance of the GALK1 enzyme, by facilitating the binding of a currently unidentified nuclear protein to the promoter region. Interestingly, c.-22 is located downstream of most of the transcription start sites identified by 5'-rapid amplification of cDNA ends (RACE) PCR [12]. The regulatory element situated downstream of the transcription start site has also been documented in genes which lack classical TATA box and contain CpG-rich regions with multiple transcription start sites [20].

Enzyme activity can be affected by missense mutations, resulting in altered amino acid sequence. Various missense mutations in *GALK1*, *GALT*, and *GALE *genes have been identified in patients with galactosemia. In addition to missense mutations, it was found that the -119 to -116delGTCA of the *GALT *gene reduced GALT activity by decreasing GALT transcription [21]. Moreover, the N314D substitution with a silent L218L substitution is associated with the 'Los Angeles phenotype', which shows increased GALT enzyme activity [22]. The suggested mechanism for increased activity of the LA variant was increased translation rates, resulting from a favorable codon bias for the mutated codon. Thus, various mechanisms, including amino acid sequence change, transcriptional regulation, and translational regulation, seem to affect the activity of enzymes involved in galactose metabolism.

The GALK1 activity of human red blood cells is only about one tenth of that of GALT, and the galactose-1-phosphate in red blood cells is a minor contributor to the total blood galactose [23,24]. These findings may suggest that elevated GALK1 activity, with normal GALT activity, has little influence on the increase of galactose-1-phosphate in erythrocytes. However, according to a study conducted on galactose metabolism in intact erythrocytes [10], newborns with mean GALK1 levels 3.4 times higher than adults also showed mean galactose-1-phosphate levels which were 3.5 times higher, indicating that transient elevation of GALK1 activity in normal newborns could result in an increase of galactose-1-phosphate. In the present study, we found that the -22T>C mutation was associated with increased GALK1 activity and could increase the expression of the reporter gene. Moreover, significantly higher galactose-1-phosphate levels were detected in individuals with GALK1 containing the 22C allele, even though they did not have any abnormalities in the GALT and GALE genes. These findings lead us to speculate that elevated GALK1 activity resulting from the -22T>C mutation may result in transient accumulation of galactose-1-phosphate, which in turn might increase the chance of positive screening results for galactosemia in newborns.

Although individuals with elevated GALK1 activity do not have a clinical phenotype of classical galactosemia, the long-term accumulation of toxic galactose 1-phosphate may cause a minor degree of damage to susceptible organs, such as the ovary and brain [25]. Further studies of the identity and the function of nuclear proteins that bind to the GALK1 promoter, with c.-22T>C, will be necessary to elucidate the mechanism and clinical significance of elevated GALK1 activity.

## Conclusion

The c.-22T>C of *GALK1 *gene was frequently observed in subjects with increased GALK1 activity and this mutation is thought to be associated with elevated GALK1 activity probably through promoting *GALK1 *expression via enhanced binding of as yet unidentified nuclear protein.

## Competing interests

The authors declare that they have no competing interests.

## Authors' contributions

JS, YS elaborated the design of the study, obtained funding, and critically revised and approved manuscript. HP, KUP, JQK, JS collected blood samples and performed PCR-direct sequencing and PCR-RFLP analysis. HP, KUP performed statistical analysis. YK, YS conducted the dual luciferase reporter assay and EMSA.

## Pre-publication history

The pre-publication history for this paper can be accessed here:


